# Effects of Deep Tillage on Wheat Regarding Soil Fertility and Rhizosphere Microbial Community

**DOI:** 10.3390/microorganisms12081638

**Published:** 2024-08-10

**Authors:** Junkang Sui, Chenyu Wang, Changqing Ren, Feifan Hou, Yuxuan Zhang, Xueting Shang, Qiqi Zhao, Xuewen Hua, Xunli Liu, Hengjia Zhang

**Affiliations:** 1 College of Agriculture and Biology, Liaocheng University, Liaocheng 252000, China; wcy20031214@163.com (C.W.); hff050129@163.com (F.H.); zyx004724@163.com (Y.Z.); sxt031216@163.com (X.S.); tavia123456@163.com (Q.Z.); huaxuewen@lcu.edu.cn (X.H.); 2 Liaocheng Science and Technology Bureau, Liaocheng 252000, China; lcskjjnczx@lc.shandong.cn; 3 College of Forestry, Shandong Agricultural University, Tai’an 271000, China; xunliliu@163.com

**Keywords:** deep tillage, rhizosphere, soil fertility, microbial community

## Abstract

Wheat production is intrinsically linked to global food security. However, wheat cultivation is constrained by the progressive degradation of soil conditions resulting from the continuous application of fertilizers. This study aimed to examine the effects of deep tillage on rhizosphere soil microbial communities and their potential role in improving soil quality, given that the specific mechanisms driving these observed benefits remain unclear. Soil fertility in this research was evaluated through the analysis of various soil parameters, including total nitrogen, total phosphorus, total potassium, available phosphorus, and available potassium, among others. The high-throughput sequencing technique was utilized to examine the rhizosphere microbial community associated with deep tillage wheat. The findings indicated that deep tillage cultivation of wheat led to reduced fertility levels in the 0–20 cm soil layer in comparison with non-deep tillage cultivation. A sequencing analysis indicated that Acidobacteria and Proteobacteria are the dominant bacterial phyla, with Proteobacteria being significantly more abundant in the deep tillage group. The dominant fungal phyla identified were Ascomycota, Mortierellomycota, and Basidiomycota. Among bacterial genera, *Arthrobacter*, *Bacillus*, and *Nocardioides* were predominant, with *Arthrobacter* showing a significantly higher presence in the deep tillage group. The predominant fungal genera included *Mortierella*, *Alternaria*, *Schizothecium*, and *Cladosporium*. Deep tillage cultivation has the potential to enhance soil quality and boost crop productivity through the modulation of soil microbial community structure.

## 1. Introduction

The cultivation of the wheat plant (*Triticum aestivum* L.) is globally widespread, with an annual production exceeding 800 million tons, of which China contributes approximately 137 million tons (http://www.fao.org/faostat/, accessed on 5 July 2024). Wheat accounts for approximately 20% of the dietary calories and protein consumed worldwide [1]. In certain regions, the application rate of fertilizer during wheat cultivation exceeds the necessary amount [2]. Increasing the nitrogen application rate has been shown to enhance the protein and constituent content in wheat. Excessive nitrogen application has been shown to decrease utilization efficiency and increase the potential for environmental contamination [3]. The long-term use of inorganic fertilizers to enhance soil fertility for crop production has been found to lead to soil acidity, reduced soil organic matter, and physical soil degradation [4]. A large amount of fertility has been left in the soil above the plough bottom for years. The accumulation of excess fertility in the soil profile over time necessitates the implementation of new tillage practices based on traditional planting methods to enhance soil quality and optimize wheat yield. Various studies have indicated that altering tillage methods can effectively adjust the interplay between soil water, fertilizer, gas, and heat, thereby resolving conflicts between crops and the soil environment, minimizing soil nutrient and water loss, and enhancing the normal growth and development of crops [5]. Deep tillage has been identified as a successful strategy for enhancing soil properties and optimizing nutrient utilization [6,7]. 

The conventional practice of continuous rotary tillage may result in the formation of a shallow plough layer, hardening of the plough bottom, accumulation of soil nutrients in the surface layer, and diminished fertilizer supply capacity for crops during the later stages of growth [8]. In contrast, deep tillage offers the potential to disrupt the hardened plough bottom, enhance soil water permeability, and facilitate a thorough mixing of nutrients throughout the entire plough layer. This, in turn, can mitigate the constraints imposed by soil compaction on the availability of nutrients [9]. Additionally, deep tillage is advantageous for soil turning, loosening, mixing, and breaking, with the added benefit of potentially increasing crop yields through reasonable deep tillage [10]. Deep tillage is typically conducted once to improve subsoil loosening, water infiltration, and root penetration [11]. This process allows for enhanced nutrient absorption by crop roots, although it may also lead to increased nitrogen loss [12]. The implementation of deep tillage to a depth of 30 cm resulted in an increase in total soil nitrogen content within the 0–40 cm soil layer, facilitating improved distribution of crop roots, enhanced water efficiency, and ultimately higher grain yields. The rotational tillage approach, involving deep tillage during the wheat season in the initial year followed by no tillage in the subsequent two years, contributed to the enhancement of stability and sustainability in winter wheat productivity [13].

In agricultural soils, microorganisms play a central role in ecological function, biological stability, and soil quality [14]. Microbes play an increasingly important role in the ecology and biological stability of agricultural soils. A variety of factors affect soil microbes, including agricultural management and land use [15]. One of the key microhabitats for plant microbiota is the rhizosphere, which serves as the boundary between the roots and soil [16]. Within the rhizosphere, free-living microbial communities engage in mutualistic interactions with the plant [17], while soil microbial communities exhibit rapid responses to stress, leading to shifts in dominant microorganism populations [18]. Deep tillage practices have a significant impact on soil microorganisms [19], affecting the patterns of microbial networks in varying ways. Furthermore, deep tillage may enhance bacterial network complexity and stability by homogenizing bacterial communities [20]. Research has shown that deep tillage can create a more favorable soil environment and a more stable soil microbial community structure [21]. As a result of deep tillage cultivation, the rhizosphere environment changes, resulting in diverse bacteria community and plant growth-promoting species being added to the ecosystem [22]. Deep tillage increased the relative abundances of Acidobacteria and Gemmatimonadetes and enhanced their amino acid metabolism, resulting in higher soil dissolved organic carbon and dissolved nitrogen contents [23]. Rotary tillage tends to complicate bacterial networks, whereas deeper tillage simplifies fungal networks [24].

This study examined the impact of deep tillage on the composition and structure of the wheat rhizosphere microbial community and elucidated the underlying mechanisms through which deep tillage affects wheat planting.

## 2. Materials and Methods

### 2.1. Study Site and Experimental Design

The research was conducted in Liaocheng, China. Liaocheng falls within the warm temperate monsoon climate zone, characterized by a semi-dry continental climate. The region experiences suitable climatic conditions, including ample sunlight with annual sunshine time ranging from 2463.0 to 2741.8 hours. The average temperature in Liaocheng ranges from 12.8 to 13.4 °C, with annual precipitation levels ranging between 567.7 and 637.3 mm and average relative humidity ranging 56–68%. The frost-free period spans approximately 200 days, characterized by prevailing southerly and slightly southerly winds. It is recommended to perform deep tillage of the soil to a depth of 30 cm prior to sowing wheat; meanwhile, the control group was performed up to a depth of 15 cm, as these were the common depths for rotary tillage in October 2022. Effective water and fertilizer management practices should be implemented throughout the wheat-growing period.

In October 2023, we established the experimental setup on land leased from Liaocheng Chuangju Fengwanjiang Agricultural Technology Development Co., Ltd. (Liaocheng, China) (115.77° E, 36.53° N) in the Dongchangfu district of Liaocheng, China. Each treatment area was oriented approximately along a south–north axis and configured in a rectangular shape measuring 20 m by 60 m. The experimental design employed a block layout. Each plot was mechanically cultivated to ensure the elimination of ridges. The YJ treatment group and the control (CK) group were separated by a distance of 20 m. Winter wheat (*Triticum aestivum* L.) cultivar ‘Jimai 22’ was sown in October 2023 and harvested in June 2023.

### 2.2. Sampling and Soil Fertility Measurement

In May 2023, root samples were collected from a depth of 15–20 cm. Excess bulk soil was removed, and the soil adhering to the roots was designated as rhizosphere soil. Simple random sampling was employed to obtain five replicates for each group. Samples of the rhizosphere soil from deep tillage (DT) wheat and non-deep tillage wheat (CK) were collected for soil fertility analysis and high-throughput sequencing, respectively, with one sample preserved at −80 °C. The soil total nitrogen (TN) content was analyzed through sulfuric acid digestion and Kjeldahl nitrogen determination [25]. Total phosphorus (TP) content in the soil was determined using NaOH alkali melting-molybdenum-antimony spectrophotometry. The total potassium (TK) content was measured through NaOH alkali fusion-flame photometry. The available phosphorus (AP) content in the soil was determined through sodium bicarbonate/sodium fluoride hydrochloric acid extraction and the molybdenum-antimony colorimetric method [26,27]. Quick available potassium (AK) content was analyzed through ammonium acetate extraction flame photometry [28]. The concentrations of nitrate nitrogen (NN) and ammonium nitrogen (AN) in soil were quantified through potassium chloride solution extraction and dual-wavelength colorimetry and potassium chloride solution extraction and indophenol blue colorimetry, respectively [29]. Soil organic matter (organic carbon) (OC) content was assessed utilizing the potassium dichromate volumetric method with external heating [30]. Soil microbial biomass carbon (MBC) content was determined through chloroform fumigation extraction [31].

### 2.3. DNA Extraction and PCR Amplification

Total microbial genomic DNA was extracted from soil samples using the E.Z.N.A.^®^ soil DNA Kit (Omega Bio-tek, Norcross, GA, USA) according to the manufacturer’s instructions. The quality and concentration of DNA were determined by 1.0% agarose gel electrophoresis and a NanoDrop^®^ ND-2000 spectrophotometer (Thermo Scientific Inc., Waltham, MA, USA), and the samples were kept at −80 °C prior to further use. The hypervariable region V3-V4 of the bacterial 16S rRNA gene were amplified with primer pairs 338F (5′-ACTCCTACGGGAGGCAGCAG-3′) and 806R(5′-GGACTACHVGGGTWTCTAAT-3′) [32] by an ABI GeneAmp^®^ 9700 PCR thermocycler (ABI, Los Angeles, CA, USA). The hypervariable region of the ITS rRNA gene were amplified with primer pairs ITS1F (5′-CTTGGTCATTTAGAGGAAGTAA-3′) and ITS2R (5′-GCTGCGTTCTTCATCGATGC-3′) [33]. The PCR reaction mixture, including 4 μL of 5 × Fast Pfu buffer, 2 μL of 2.5 mM dNTPs, 0.8 μL of each primer (5 μM), 0.4 μL of Fast Pfu polymerase, 10 ng of template DNA, and ddH_2_O, up to a final volume of 20 µL. The PCR amplification cycling conditions were as follows: initial denaturation at 95 °C for 3 min, followed by 27 cycles of denaturing at 95 °C for 30 s, annealing at 55 °C for 30 s and extension at 72 °C for 45 s, and single extension at 72 °C for 10 min, ending at 4 °C. All samples were amplified in triplicate. The PCR product was extracted from 2% agarose gel and purified using the AxyPrep DNA gel extraction kit (Axygen Biosciences, Union City, CA, USA) according to the manufacturer’s instructions and quantified using a Quantus™ fluorometer (Promega, Madison, WI, USA).

### 2.4. Illumina MiSeq Sequencing

Purified amplicons were pooled in equimolar amounts and paired-end sequenced on an Illumina MiSeq PE300 platform (Illumina, San Diego, CA, USA) according to the standard protocols by Majorbio Bio-Pharm Technology Co., Ltd. (Shanghai, China). The raw sequencing reads were deposited into the NCBI database with the BioProject accession number PRJNA1138004.

### 2.5. Data Processing

Raw FASTQ files were de-multiplexed using an in-house perl script and then quality-filtered by fastp version 0.19.6 [34] and merged by FLASH version 1.2.7 [35] with the following criteria: (i) The 300 bp reads were truncated at any site receiving an average quality score of <20 over a 50 bp sliding window, and truncated reads that were shorter than 50 bp were discarded. Reads containing ambiguous characters were also discarded; (ii) Only overlapping sequences longer than 10 bp were assembled according to their overlapped sequence. The maximum mismatch ratio of the overlap region was 0.2. Reads that could not be assembled were discarded; (iii) Samples were distinguished according to the barcode and primers, and the sequence direction was adjusted. Exact barcode matching was performed, and there was a 2-nucleotide mismatch in the primer matching. Then, the optimized sequences were clustered into operational taxonomic units (OTUs) using UPARSE 7.1 [36,37] with a 97% sequence similarity level. The most abundant sequence for each OTU was selected as a representative sequence. To minimize the effects of sequencing depth on alpha and beta diversity measurements, the number of 16S rRNA gene sequences from each sample were rarefied, which still yielded an average Good’s coverage of 99%, respectively.

### 2.6. Statistical Analysis

The bioinformatic analysis of the soil microbiota was carried out using the Majorbio Cloud platform (https://cloud.majorbio.com, accessed on 21 May 2024). Based on the OTUs information, rarefaction curves and alpha diversity indices including the observed OTUs, Chao1 richness, Shannon index, and Good’s coverage were calculated with Mothur v1.30.1 [38]. The similarity among the microbial communities in different samples was determined by a principal coordinate analysis (PCoA) based on Bray–Curtis dissimilarity using the Vegan v2.5-3 package. The PERMANOVA test was used to assess the percentage of variation explained by the treatment along with its statistical significance using the Vegan v2.5-3 package. The linear discriminant analysis (LDA) effect size (LEfSe) [39] (http://huttenhower.sph.harvard.edu/LEfSe, accessed on 24 May 2024) was performed to identify the significantly abundant taxa (phylum to genera) of bacteria among the different groups (LDA score > 2, *p* < 0.05).

The results are presented as the mean ± standard deviation (SD). Significant differences in soil fertility, diversity, and richness indices between the DT and CK groups were identified using a one-way ANOVA test, followed by Duncan’s multiple range tests, with significance levels set at *p* < 0.05. All statistical analyses were conducted using SAS, version 9 (SAS Institute Inc., Cary, NC, USA).

## 3. Results

### 3.1. Changes in Edaphic Properties

The findings of the soil fertility factor analysis indicated that the content of total nitrogen (TN), total phosphorus (TP), total potassium (TK), available phosphorus (AP), available potassium (AK), organic matter (OC), and microbial biomass carbon (MBC) were significantly (*p* < 0.05) reduced in the deep tillage cultivation group (DT) compared with the control group (CK). However, there was no significant disparity in nitrate nitrogen (NN) and ammonium nitrogen (AN) content between the two groups (Table 1).

### 3.2. Sequencing Quality Evaluation

**Figure 1:**
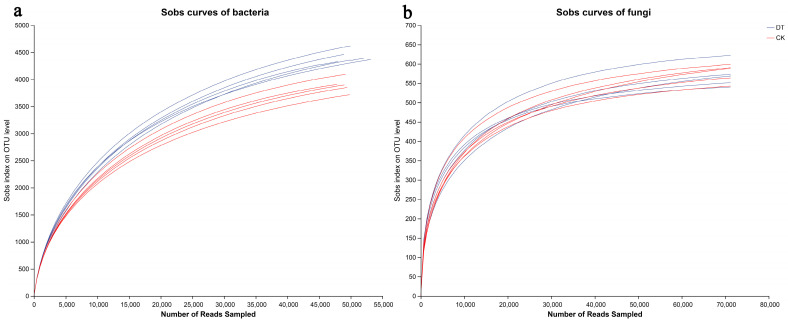
Bacterial (**a**) and fungal sobs curves (**b**) were examined to assess the impact of a 3% dissimilarity cutoff on the identification of uncovered operational taxonomic units (OTUs). The abbreviation “DT” refers to deep tillage-cultivated wheat rhizosphere soil group, while “CK” represents the non-deep tillage-cultivated wheat rhizosphere soil group.

**Figure 2:**
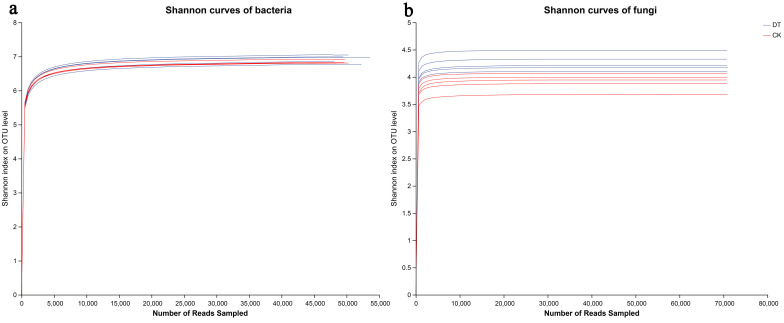
Bacterial and fungal Shannon curves (**a**,**b**) were examined to assess the impact of a 3% dissimilarity cutoff on the identification of uncovered operational taxonomic units (OTUs). The abbreviation “DT” refers to the deep tillage-cultivated wheat rhizosphere soil group, while “CK” represents the non-deep tillage-cultivated wheat rhizosphere soil group.

According to the sequencing data, a total of 80,171 and 79,701 bacterial 16S rDNA sequences were acquired for the DT and CK groups, respectively, in addition to 84,234 and 86,713 fungal ITS sequences for the DT and CK groups. By applying a clustering dissimilarity threshold of 3%, the reads were assigned to different operational taxonomic units (OTUs). The Sobs diversity rarefaction curves for bacterial and fungal communities did not plateau at a distance of 0.03 (Figure 1), suggesting that the sequencing data did not fully capture all community diversity. However, the integration of the Shannon diversity index and rarefaction curves provided a more thorough assessment of community diversity (Figure 2). Based on the Shannon diversity curves, it can be observed that as the quantity of reads grew, the curves reached a plateau, suggesting that an ample amount of data had been gathered for the analysis of communities.

The analysis of diversity and richness indices in the soil samples (Table 2) revealed that the DT and CK groups exhibited similar levels of bacterial community richness and diversity. However, the ACE, Chao, and Sobs values, which serve as indicators of species richness, were relatively higher in the rhizosphere bacterial community in the DT group. The Shannon and Simpson diversity indices did not show significant differences.

In terms of fungal community diversity and richness, the ACE, Chao, and Sobs values showed slightly lower values in the DT group than those in the CK group but did not exhibit significant differences. The lower Simpson index observed in the DT group indicated a higher level of diversity; at the same time, the higher Shannon index observed in the DT group indicated a higher level of diversity. These results of microbial community richness and diversity indices reveal that the CK group exhibited a greater abundance and diversity of fungal communities compared with the DT group. Conversely, the DT group enhanced a higher abundance of bacterial communities compared with the CK group.

**Figure 3:**
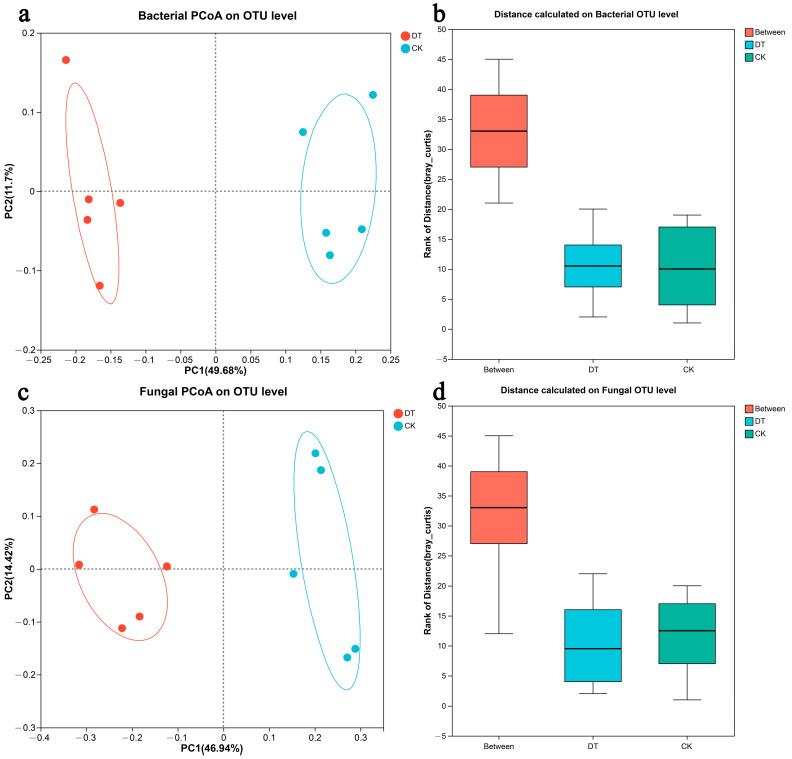
PCoA analysis and ANOSIM analysis of deep tillage and non-deep tillage cultivation of wheat rhizosphere soil microbes. The abbreviation “DT” refers to the deep tillage-cultivated wheat rhizosphere soil group, while “CK” represents the non-deep tillage-cultivated wheat rhizosphere soil group. (**a**, PCoA analysis of DT. **b**, PCoA analysis of CK. **c**, ANOSIM analysis of DT. **d**, ANOSIM analysi of CK.)

By conducting an inter-group comparative analysis of species diversity between different microbial communities, the similarities or differences in community composition between samples in different groups were explored. In the PCoA analysis of bacteria and fungi, the percentage represents the explanation value of the main coordinate axis for the difference in sample composition, which indicated a well-defined and representative ordination. The distance between the DT group and the CK group was found to be greater than among intragroup samples, suggesting a high level of community aggregation and dispersion (Figure 3a,c). An ANOSIM analysis further confirmed that the dissimilarity between the two groups was significantly higher than the dissimilarity within each group (Figure 3b,d).

### 3.3. Microbial Community Composition and Structure

**Figure 4:**
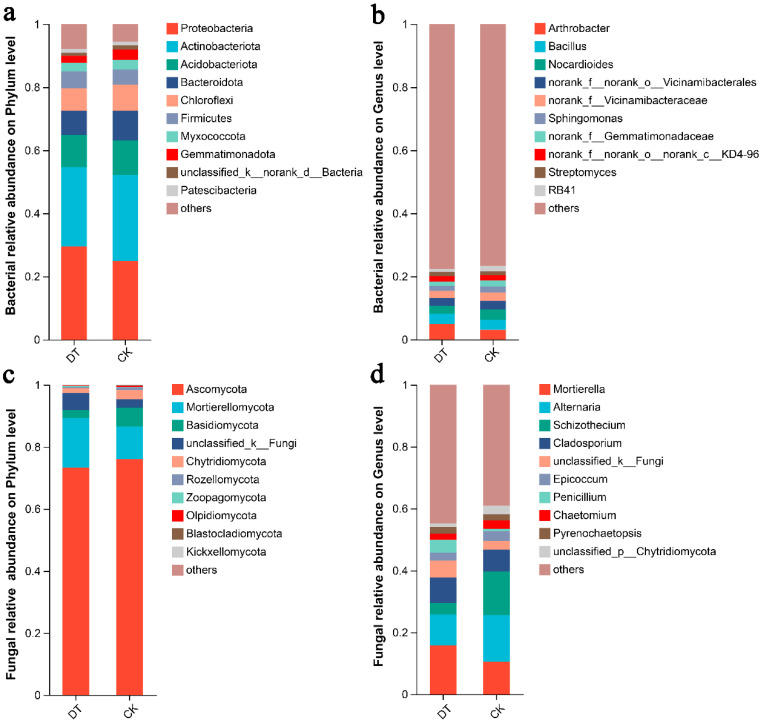
Communities of bacteria and fungi in the study group. (**a**) Relative abundances of bacteria at the phylum level; (**b**) relative abundances of bacteria at the genus level; (**c**) relative abundances of fungi at the phylum level; (**d**) relative abundances of fungi at the genus level. The relative abundances of major genera are illustrated in stacked bar graphs. The abbreviation “DT” refers to the deep tillage-cultivated wheat rhizosphere soil group, while “CK” represents the non-deep tillage-cultivated wheat rhizosphere soil group.

Sequence classification was performed using the Mothur program. At the phylum level, the predominant rhizosphere soil bacteria identified were Pseudomonadota, Actinomycetota, Acidobacteriota, Bacteroidota, Chloroflexota, and Bacillota, collectively representing over 85% of the total bacterial phyla abundance (Figure 4a). The Proteobacteria phylum constituted 29.47% and 24.94% of the DT and CK groups, respectively. Actinobacteriota constituted 25.11% and 27.18% of the DT and CK groups, respectively. The Acidobacteriota phylum constituted 10.28% and 11.02% of the DT and CK groups, respectively.

The predominant phyla of fungi were Ascomycota, Mortierellomycota, Basidiomycota, and Chytridiomycota, collectively representing over 91% of the total fungal phyla abundance (Figure 4c). The Ascomycota phylum constituted 73.34% and 76.08% of the DT and CK groups, respectively, representing the largest phylum at the phylum level. Mortierellomycota constituted 15.91% and 10.56% of the DT and CK group, respectively. Basidiomycota constituted 2.67% and 5.94% of the DT and CK group, respectively.

Overall, the bacterial compositions at the genus level were similar between the two groups, although individual genera were distributed differently between the two groups. Excluding unidentified genera, the genus of *Arthrobacter*, *Bacillus*, and *Nocardioides* are the predominant genus (Figure 4b). The relative abundance of *Arthrobacter* was 4.99% and 3.08% in the DT and CK groups, respectively. *Bacillus* had a relative abundance of 3.24% and 3.12% in the DT and CK groups, respectively. *Nocardioides* had a relative abundance of 2.44% and 3.30% in the DT and CK groups, respectively.

The fungal compositions at the genus level showed more obvious differences. The genera of *Mortierella*, *Alternaria*, *Schizothecium*, and *Cladosporium* are the predominant genus (Figure 4d). The relative abundance of *Mortierella* was 15.82% and 10.52% in the DT and CK groups, respectively. The relative abundance of *Alternaria* was 10.01% and 15.04% in the DT and CK groups, respectively. The relative abundance of *Schizothecium* was 3.63% and 14.03% in the DT and CK groups, respectively. The relative abundance of *Cladosporium* was 8.31% and 7.14% in the DT and CK groups, respectively.

**Figure 5:**
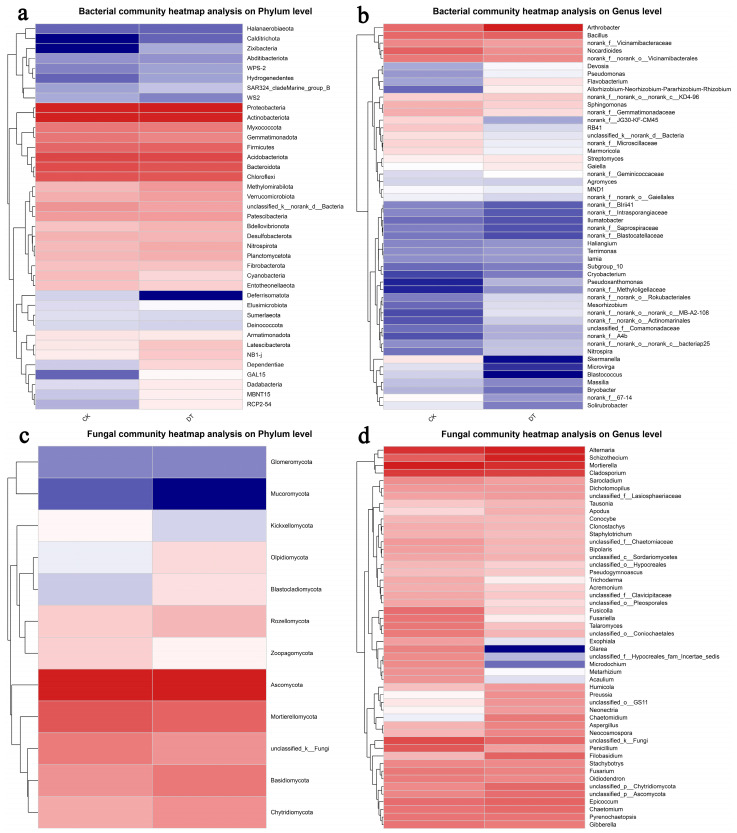
Hierarchical clustering of bacterial and fungal distributions. (**a**) Relative abundances of bacteria at the phylum level; (**b**) relative abundances of bacteria at the genus level; (**c**) relative abundances of fungi at the phylum level; (**d**) relative abundances of fungi at the genus level. The abbreviation “DT” refers to the deep tillage-cultivated wheat rhizosphere soil group, while “CK” represents the non-deep tillage-cultivated wheat rhizosphere soil group.

The results of the hierarchical clustering of bacterial and fungal distributions as shown in the heatmap provided confirmation to the community bar plot that Pseudomonadota, Actinomycetota, Acidobacteriota, Bacteroidota, Chloroflexota, and Bacillota are the main components with higher proportions in the bacteria phylum in both groups. Moreover, the CK group has very little content of Calditrichota and Zixibacteria, and the DT group has very little content of Deferrisomatota (Figure 5a). The genera of *Arthrobacter*, *Bacillus*, and *Nocardioides* are the predominant genera, with higher relative abundance in the bacteria genera in both groups. On the other hand, the genus of *Skermanella* and *Blastococcus* showed particularly low relative content in the DT group, and the genus of *Pseudoxanthomonas* showed particularly low relative content in the CK group (Figure 5b).

The Ascomycota and Mortierellomycota phyla are the two fungal phyla with the highest content. Furthermore, the Mucoromycota phylum showed a particularly low relative content in the CK group (Figure 5c). The bacterial genera of *Alternaria*, *Schizothecium*, *Mortierella*, and *Cladosporium* have larger relative content on genus level than others in both groups. On the other hand, the bacterial genera of *Glarea*, *Microdochium*, and *Metarhizium* have particularly low relative content in the CK group, and the genera of *Preussia*, *Neonectria*, and *Chaetomidium* have particular low relative content in the DT group (Figure 5d).

**Figure 6:**
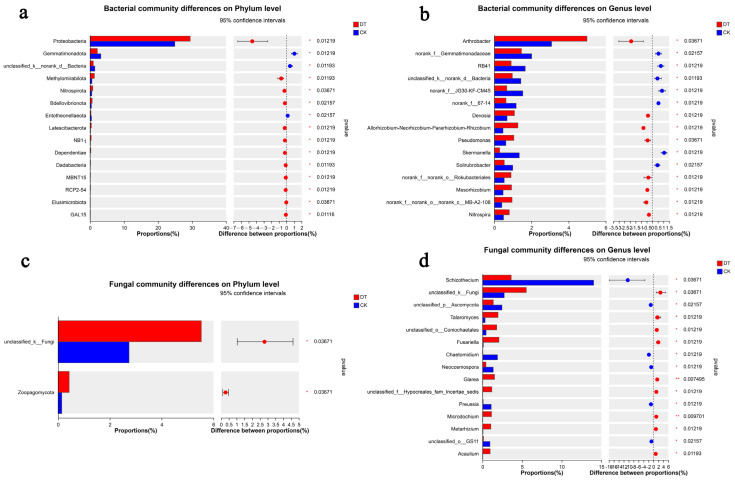
Significant test of differences between the two groups. (**a**) Significant differences in bacteria at the phylum level; (**b**) significant differences in bacteria at the genus level; (**c**) significant differences in fungi at the phylum level; (**d**) significant differences in fungi at the genus level. The abbreviation “DT” refers to the deep tillage-cultivated wheat rhizosphere soil group, while “CK” represents the non-deep tillage-cultivated wheat rhizosphere soil group.

The different microorganisms may be key species in responding to environmental changes. At the bacterial phylum level, the relative abundances of Pseudomonadota, Methylomirabilota, and Nitrospirota were significantly higher in the DT group than in the CK group (*p* < 0.05), but the relative abundances of Gemmatimonadota and Latescibacterota were significantly higher in the CK group than in the DT group (*p* < 0.05) (Figure 6a). At the bacterial genus level, the relative abundances of *Arthrobacter*, *Devosia*, and *Pseudomonas* were significantly higher in the DT group (*p* < 0.05). On the other hand, the relative abundances of *Skermanella* and *Solirubrobacter* were significantly higher in the CK group (*p* < 0.05) (Figure 6b).

At the fungal phylum level, the relative abundance of zoopagomycota was significantly higher in the DT group than in the CK group (*p* < 0.05) (Figure 6c). At the fungal genus level, the relative abundances of *Talaromyces* and *Fusariella* were significantly higher (*p* < 0.05) in the DT group, as was also the case for *Glarea* and *Microdochium* (*p* < 0.01). On the other hand, the relative abundances of *Schizothecium*, *Chaetomidium*, *Neocosmospora*, and *Preussia* were significantly lower in the DT group (*p* < 0.05) (Figure 6d).

**Figure 7:**
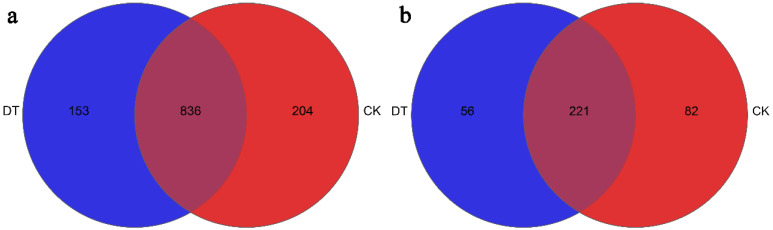
Unique and shared genera of (**a**) bacteria and (**b**) fungi for the two groups in Venn diagram form. The abbreviation “DT” refers to the deep tillage-cultivated wheat rhizosphere soil group, while “CK” represents the non-deep tillage-cultivated wheat rhizosphere soil group. We analyzed three replicates for every treatment.

The Venn diagram in Figure 7 illustrates the presence of bacterial and fungal genus in both study groups. The number of bacterial genera observed in the DT treatment was 989, while the CK treatment had 1040 genera. Among these, 836 were shared between the DT and CK groups. Notably, three primary genera (*Arthrobacter*, *Bacillus*, and *Nocardioides*) were found in both groups, while *Pedosphaera*, *Gemmata*, and *Epulopiscium* were exclusively present in the DT group. Conversely, *Larkinella*, *Prosthecomicrobium*, and *Sporobacter* were only detected in the CK group.

A total of 277 fungal genus were identified in the DT group, while the CK group exhibited 303 genera. Among these, 221 genera were shared between the two groups, with *Mortierella*, *Alternaria*, *Schizothecium*, and *Cladosporium* being the common fungal taxa. Conversely, *Geastrum*, *Rhodosporidiobolus*, *Phialocephala*, and *Pseudocosmospora* were exclusively detected in the DT group, whereas *Ganoderma*, *Niesslia*, *Exserohilum*, and *Keissleriella* were solely observed in the CK group.

**Figure 8:**
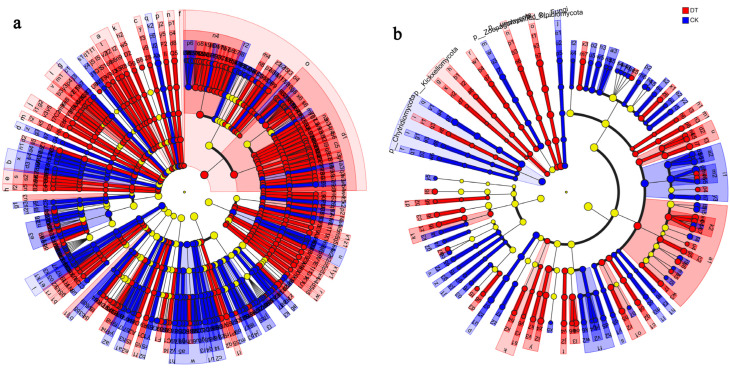
Discriminant analysis of muti-level species differences obtained through LEfSe analysis. (**a**) Differences in bacterial multi-level species in the DT and CK groups. (**b**) Differences in fungal multi-level species in the DT and CK groups. Differently colored nodes represent microbial communities that are significantly enriched in their corresponding groups and have a significant impact on inter-group differences.

The present study employed an LEfSe analysis to perform difference tests at multiple levels in the rhizospheres of deep tillage-cultivated wheat and non-deep tillage-cultivated wheat. It was found that *Arthrobacter*, *Flavobacterium*, *Pseudomonas*, *Mesorhizobium*, *Devosia*, and *Pseudoxanthomonas* were specific bacterial genera in the DT group, and *Skermanella*, *Blastococcus*, *Microvirga*, *Solirubrobacter*, *Confluentibacter*, and *Rubrobacter* were specific bacterial genera in the CK group (Figure 8a). The *Talaromyces*, *Glarea*, *Microdochium*, *Metarhizium*, *Acaulium*, and *Exophiala* were specific fungal genera in the DT group, and *Schizothecium*, *Chaetomidium*, *Preussia*, *Neocosmospora*, *Neonectria*, and *Humicola* were specific fungal genera in the CK group (Figure 8b).

## 4. Discussion

The prevalent agricultural technique of returning straw to the field, when combined with deep tillage, has been demonstrated to effectively alleviate soil compaction and augment soil depth [40]. Moreover, deep tillage enhances crop yields by integrating straw and fertilizer from the surface layer into the deeper soil strata, thereby providing nutrients to both the deeper soil layers and the crops [10,41]. The analysis of soil fertility data demonstrates that deep tillage (DT) in wheat cultivation leads to reduced fertility levels in the 0–20 cm soil layer when compared with rotary tillage (CK). These results are consistent with prior research, which suggests that rotary tillage during the wheat season enhances soil nutrient content within the 0–20 cm layer, while deep tillage improves nutrient levels in the 20–40 cm layer [42]. Regular implementation of rotary tillage promotes the accumulation of plant residues, straw, fertilizers, and other nutrients on the soil surface, thereby enhancing nutrient availability in the upper soil layers [43]. Soil fertility is related to soil microbial communities, and consistent with previous studies, soil fertility affects soil microbial growth; soil microbial richness also affects soil fertility [44].

The sequencing has yielded a substantial dataset for the analysis of microbial communities. The results indicate that deep tillage significantly increased species richness within the rhizosphere bacterial community, whereas non-deep tillage fostered greater diversity within the rhizosphere fungal community. Several studies investigating various tillage systems for soybean cultivation have demonstrated that both fungal and bacterial communities are influenced by the tillage practices employed. Furthermore, the soil microbial community and its functional attributes may respond to different management strategies and land use practices [45]. The analysis of microbial network patterns under different tillage methods indicates that deep tillage complicates bacterial network structures but simplifies fungal networks [24].

The distribution of the microbial community at the operational taxonomic unit (OTU) level was examined using β-diversity metrics, specifically through the use of a principal coordinates analysis (PCoA). From the standpoint of soil microbial community succession, the PCoA ordination plots indicated substantial impacts on microbial community structure following deep tillage cultivation. This method is frequently employed to illustrate the similarities and differences within microbial communities [46,47]. The ANOSIM analysis further substantiated that the dissimilarity between the rhizosphere soil microbial communities under deep tillage and non-deep tillage conditions was significantly greater than the dissimilarity observed within each respective group. Typically, the ANOSIM analysis is employed to elucidate differences both between and within groups [48].

The phyla Pseudomonadota, Actinomycetota, and Acidobacteriota predominated in both the DT and CK groups. These phyla are prevalent in most soils and have been recognized for their significance as antagonistic microorganisms; at the same time, studies have indicated that Pseudomonadota play a direct role in the soil carbon cycle [49]. Some studies have indicated that Proteobacteria are the most abundant species in soil [50]. Notably, the abundance of Pseudomonadota was significantly higher in the DT group, where it has consistently been the dominant bacterial phylum [51], particularly in the root zone and rhizosphere soil [52]. Acidobacteriota are specifically abundant in soil ecosystems [53]. Furthermore, research has demonstrated their involvement in the degradation of plant and microbial polysaccharides, as well as their association with soil nitrogen availability [54].

The sequencing analysis revealed that the genera *Arthrobacter*, *Bacillus*, and *Nocardioides* were predominant in both the DT and CK rhizospheres, consistent with their established prevalence in such environments. Notably, the relative abundances of *Arthrobacter* and *Pseudomonas* were significantly higher in the DT group. *Arthrobacter*, in particular, is a heterogeneous genus of bacterial species that is widely distributed in various environments, especially in soil [55,56]. Representatives of the Arthrobacter genus have been reported to degrade numerous organic pollutants [57] and are also known to produce enzymes such as amylase, lipase, and protease [58]. The *Bacillus* genus has demonstrated a range of beneficial effects on soil and plants, including the production of various biological enzymes that prevent and control plant pathogens [59,60]. Additionally, several members of the *Pseudomonas* genus, such as *Pseudomonas fluorescens*, *Pseudomonas putida*, and *Pseudomonas aeruginosa*, have been identified as highly effective in promoting plant growth and providing biocontrol [61].

In this study, Ascomycota, Mortierellomycota, Basidiomycota, and Chytridiomycota emerged as the most prevalent fungal phyla, corroborating findings from previous research [52,62,63] on the dominant fungal phyla in soil. Ascomycota are greatly affected by plant degradation and straw residues, and their abundance increases when nutrients are added [64]. Notably, the relative abundance of Zoopagomycota was significantly higher in the DT group. Members of Zoopagomycota are predominantly associated with animals, functioning either as commensals or pathogens or as mycoparasites [65]. In terms of fungal genus composition, *Mortierella*, *Alternaria*, *Schizothecium*, and Cladosporium are predominant. Mortierella, a commonly isolated filamentous fungus from the environment, has been reported to confer benefits to plants [66]. Additionally, Mortierella strains play a crucial role in bioremediation by degrading organic pollutants [67], and they have shown the capability to oxidize carbon monoxide, an inorganic compound [68]. The relative abundance of *Talaromyces* was significantly higher in the DT group at the fungal genus level. It is known that *Talaromyces* colonize and decompose leaves [69]. It has also been suggested that *Talaromyces* isolates’ efficacy is due to their ability to produce cellulose [70].

## 5. Conclusions

Deep tillage has the potential to significantly modify soil structure, offering advantages such as soil inversion, loosening, mixing, and fragmentation. Furthermore, deep tillage may contribute to influences on soil microorganism populations. This study employed high-throughput sequencing techniques to assess changes in the microbial community structure, with a primary focus on evaluating the community of bacterial and fungal genera within the soil of wheat cultivated under deep tillage conditions, as compared with non-deep tillage conditions. Our findings demonstrate that bacterial diversity and richness were elevated in the rhizosphere soils of wheat subjected to deep tillage as opposed to those under non-deep tillage conditions. Notably, we observed significant increases in the abundances of the genera *Arthrobacter*, *Devosia*, and *Pseudomonas* in deep tillage soils. Furthermore, the presence of the fungal genera *Mortierella* and *Talaromyces* was also found to be more pronounced in deep tillage soils. These may be the reasons for the improvement in the soil microbial community structure in deep tillage wheat planting. However, the functional mechanisms underlying these observations remain unclear, necessitating further research to elucidate their specific characteristics and ecological roles.

## Figures and Tables

**Table 1:** Soil fertility characteristics of the deep tillage cultivation and control groups.

	TN g/kg	TP g/kg	TK g/kg	AP mg/kg	AK mg/kg	NN mg/kg	AN mg/kg	OC g/kg	MBC mg/kg
DT	0.78 ± 0.10 ^b^	0.75 ± 0.15 ^b^	19.34 ± 0.55 ^b^	11.81 ± 2.06 ^b^	94.89 ± 11.92 ^b^	70.77 ± 6.22 ^a^	10.43 ± 1.06 ^a^	11.62 ± 1.65 ^b^	215.94 ± 13.42 ^b^
CK	1.41 ± 0.09 ^a^	1.21 ± 0.10 ^a^	21.28 ± 0.72 ^a^	24.11 ± 7.82 ^a^	180.97 ± 40.57 ^a^	49.30 ± 5.97 ^b^	13.98 ± 3.68 ^a^	23.79 ± 3.18 ^a^	571.51 ± 143.23 ^a^

The data are displayed as the mean ± standard error (SE), with statistical significance set at *p* < 0.05, denoted by lowercase superscript letters within the same column. The term “DT” denotes the deep tillage-cultivated wheat rhizosphere soil group, whereas “CK” signifies the non-deep tillage-cultivated wheat rhizosphere soil group.

**Table 2:** The diversity and richness indices of bacterial and fungal communities in perennial poplar big trees and poplar seedlings in soil with continuous cropping.

	Sample	ACE	Chao	Sobs	Simpson	Shannon	Coverage
Bacterial	DT	5228.87 ± 182.36 ^a^	4981.95 ± 164.23 ^a^	4378.20 ± 127.97 ^a^	0.0048 ± 0.0013 ^a^	6.92 ± 0.12 ^a^	0.9775
	CK	4552.78 ± 119.48 ^b^	4328.56 ± 107.71 ^b^	3866.40 ± 144.60 ^b^	0.0036 ± 0.0004 ^a^	6.83 ± 0.05 ^a^	0.9815
Fungal	DT	599.85 ± 34.66 ^a^	600.35 ± 29.80 ^a^	570.80 ± 31.52 ^a^	0.035 ± 0.008 ^b^	4.27 ± 0.15 ^a^	0.9992
	CK	614.75 ± 31.74 ^a^	610.31 ± 30.82 ^a^	576.60 ± 23.05 ^a^	0.062 ± 0.018 ^a^	3.92 ± 0.15 ^b^	0.9991

The data are displayed as the mean ± standard error (SE), with statistical significance set at *p* < 0.05, denoted by lowercase superscript letters within the same column. The term “DT” denotes the deep tillage-cultivated wheat rhizosphere soil group, whereas “CK” signifies the non-deep tillage-cultivated wheat rhizosphere soil group.

## Data Availability

The raw sequencing data were submitted to the NCBI database BioProject under accession number PRJNA1138004.
